# Analysis of urinary exosomal metabolites identifies cardiovascular risk signatures with added value to urine analysis

**DOI:** 10.1186/s12915-020-00924-y

**Published:** 2020-12-14

**Authors:** Marta Agudiez, Paula J. Martinez, Marta Martin-Lorenzo, Angeles Heredero, Aranzazu Santiago-Hernandez, Dolores Molero, Juan Manuel Garcia-Segura, Gonzalo Aldamiz-Echevarria, Gloria Alvarez-Llamas

**Affiliations:** 1 Immunology Department, IIS-Fundacion Jimenez Diaz-UAM, Madrid, Spain; 2 Cardiac Surgery Department, Fundacion Jimenez Diaz-UAM, Madrid, Spain; 3 CAI-RMN, Universidad Complutense, Madrid, Spain; 4 Department of Biochemistry and Molecular Biology, Faculty of Biology, Universidad Complutense, Madrid, Spain; 5 REDINREN, Madrid, Spain

**Keywords:** Cardiovascular risk, Exosomes, Metabolites, Urine, Biomarkers, NMR

## Abstract

**Background:**

Subclinical atherosclerosis may result in fatal cardiovascular (CV) events, but the underlying mechanisms and molecular players leading to disease are not entirely understood. Thus, novel approaches capable of identifying the factors involved in pathological progression and providing a better understanding of the subjacent mechanisms are needed. Extracellular vesicles (EVs) have been shown to have numerous biological functions, and their metabolome has recently generated interest as a source of novel biomarkers. The metabolic content of the exosomes has been so far unexplored in cardiovascular disease (CVD), and here, we developed an analytical strategy aimed at probing urinary exosomal metabolite content and its association to CV risk.

**Results:**

Direct analysis of the exosomes without metabolite extraction was evaluated by high-resolution magic angle spinning (^1^H HR-MAS). Other two methodologies for the analysis of exosomal metabolites by ^1^H NMR were set up, based on methanol or organic solvents sequential extraction. The three methods were compared in terms of the number of detected signals and signal to noise ratio (S/N). The methanol method was applied to identify altered metabolites in the urinary exosomes of subjects with programmed coronary artery by-pass grafting (CABG) versus a control group. Target mass spectrometry (MS) was also performed for differential analysis. The clinical performance of exosomal metabolites of interest in CVD was investigated, and the added value of the exosomes compared to urine analysis was evaluated.

Based on S/N ratio, simplicity, reproducibility, and quality of the spectrum, the methanol method was chosen for the study in CVD. A cardiometabolic signature composed by 4-aminohippuric acid, N-1-methylnicotinamide, and citric acid was identified in urinary exosomes. Directly in urine, 4-aminohippuric acid and citric acid do not show variation between groups and changes in N-1-methylnicotinamide are less pronounced, proving the added value of exosomes.

**Conclusions:**

We set up a novel methodology to analyze metabolic alterations in urinary exosomes and identified a cardiometabolic signature in these microvesicles. This study constitutes the first evidence of a role for the exosomal metabolism in CVD and demonstrates the possibility to evaluate the urinary exosomal metabolic content by NMR and MS.

**Supplementary Information:**

The online version contains supplementary material available at 10.1186/s12915-020-00924-y.

## Background

Despite the knowledge of traditional risk factors and the enormous efforts dedicated to improve prevention, cardiovascular disease (CVD) remains the leading cause of death worldwide [1]. Subjacent mechanisms involve multiple factors and acting molecules playing together in a complex network which is not fully understood. Thus, further knowledge of these acting molecules is needed. The metabolome represents the ultimate response of the organism to a pathophysiological condition, and the metabolites are the molecules most closely reflecting the cell phenotype. In CVD, specific metabolites have been identified in biological fluids [2–4], whole arterial tissue [5], and arterial layers at their specific location [6]. Extracellular vesicles (EVs) constitute an additional source of information. They act as biological messengers in cell-to-cell communication, RNA and protein transfer, and immune response regulation [7, 8], with a growing interest in their potential role as a therapeutic tool in tissue regeneration and as a way for drug delivery [9]. We have previously showed how urinary exosomes reflect protein changes taking place in the kidney in diabetic nephropathy, constituting an accessible source of information complementary to renal biopsy [10] and more recently in hypertensives developing albuminuria as a main CV risk factor [11]. However, the metabolome of EVs is a new focus of interest since very recently, mainly in cancer, to investigate inter-cellular communication or novel biomarkers if present in EVs isolated from biological fluids [12–14]. In CVD, the EV metabolism has been so far unexplored.

Following previous studies by our group using nuclear magnetic resonance (NMR) applied to CVD and renal disease [5, 15–18], we aimed first to develop for the first time a methodological approach to analyze the urinary exosome metabolome by NMR also compatible with LC-MS analysis. Then, we aimed to investigate the metabolome of urinary exosomes in CVD, particularly in patients undergoing coronary artery by-pass grafting (CABG) as representative population of high CV risk. Three different protocols were set up and compared, two of them based on metabolite extraction from urinary exosomes and a third one consisting in direct analysis of the exosomal pellet. The best-performing method was then applied as a screening methodology to investigate differences in patients with high CV risk. Selected metabolites were further analyzed by target mass spectrometry (MS) in selected reaction monitoring mode (SRM). Their potential in the clinic was assessed, and their added value to whole urine analysis in CVD evaluated.

## Results

### Direct analysis of the exosomal pellet by ^1^H HR-MAS versus metabolite extraction and NMR analysis

**Fig. 1:**
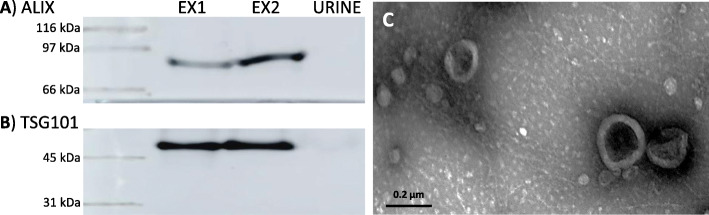
Characterization of urinary exosomes isolated from urine. **a** Western blot of Alix. **b** Western blot of TSG101. **c** Electron microscopy image. Two exosomal fractions (EX) from two different healthy controls (EX1 and EX2) were analyzed and compared to urine

Urinary exosomes were isolated by ultracentrifugation. The replacement of the sucrose solution by PBS assures the exosomal integrity while eliminating sucrose interference in the NMR spectrum. Figure 1 shows Western blot analysis of the well-known exosomal markers Alix and TSG101 and an electron microscopy image of a representative urinary exosome preparation obtained with the protocol herein described.

**Fig. 2:**
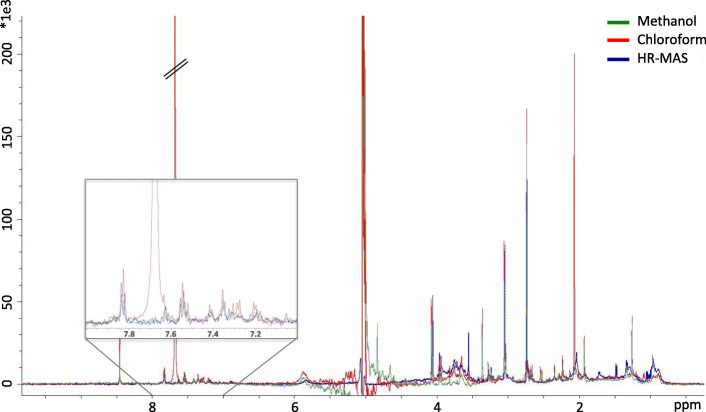
Evaluation of the three methodological approaches developed for the analysis of the exosomal metabolome. The three spectra corresponding to methanolic extraction (^1^H NMR), organic solvent extraction (^1^H NMR), and direct analysis of the exosomal pellet (without extraction) (^1^H HR-MAS) are shown overlaid. ^1^H HR-MAS spectrum was scaled by a factor of 0.1301 for proper visual comparison matching the noise level

To set up a protocol for the analysis of the exosomal metabolites by ^1^H NMR, we first optimized the extraction of the metabolites from the exosomal pellet to the liquid phase. On the one hand, a methanol-based extraction was performed, and on the other hand, a sequential extraction in organic solvents of decreasing polarity was performed (chloroform-based method). Additionally, we analyzed the exosomal pellet without metabolite extraction by ^1^H HR-MAS, testing the potential of this technique for the direct analysis of the isolated exosomes. Figure 2 shows the representative spectra obtained for the two extraction methods (methanol-based and chloroform-based) and for the direct analysis of the exosomal pellet (non-extraction method). The high intensity signal of chloroform (7.7 ppm) can be clearly seen, thus being a potential mask of lower-abundance metabolites of interest in the aromatic area of the spectra.

**Fig. 3:**
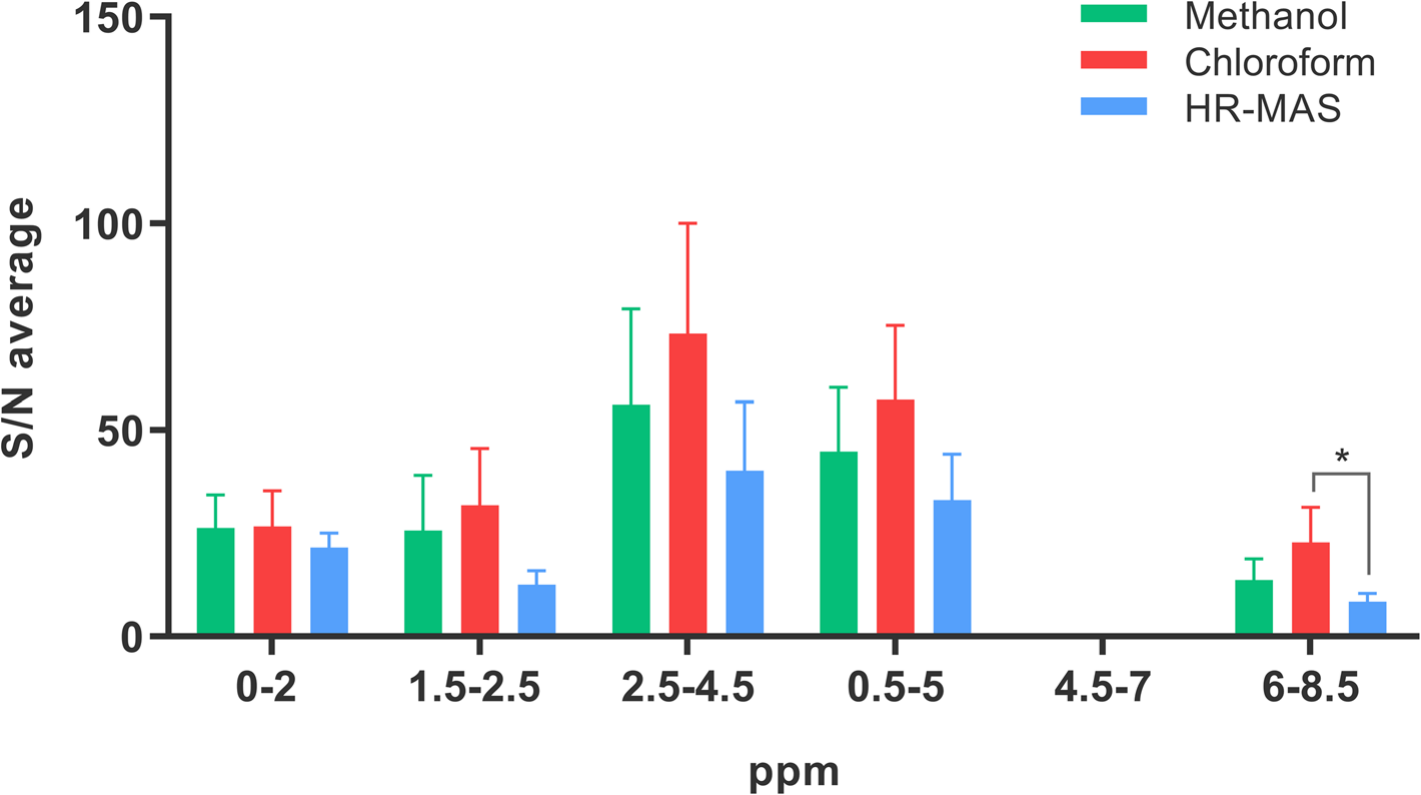
Average S/N values obtained for each region of the spectrum and calculated including all peaks detected by the three methods (methanol-based extraction, chloroform-based extraction, or direct analysis by ^1^H HR-MAS). *p value = 0.0185. The number of common signals per region was 8 signals (0–2 ppm), 5 signals (1.5–2.5 ppm), 19 signals (2.5–4.5 ppm), 29 signals (0.5–5 ppm), and 10 signals (6.5–8.5 ppm)

In an attempt to characterize each of these methods individually and estimate which metabolite classes are favored in each of them, the total number of detected peaks was calculated per method and spectral region and classified according to their intensity range (low, medium, or high) (Additional file 1: Fig. S1). For the three protocols, the highest number of detected peaks and of “high intensity” peaks are in the upfield area (0–5 ppm), while the “low intensity” peaks are grouped mainly in the aromatic and vinylic area, i.e., in the region from 5 to 10 ppm or downfield area, where the number of peaks is significantly lower than in the upfield. Besides, the number of detected peaks was calculated with different sensitivity cut-off values, i.e., 3, 5, 8, and 10 times the S/N value (Additional file 2: Fig. S2). ^1^H HR-MAS method resulted in the highest number of detected peaks with intensity values higher than 3 times S/N. However, if this cut-off value was increased, the sensitivity deeply and proportionally diminished (from 96 detected peaks to 64 when peak intensity cut-off value was 3 or 10 times S/N, respectively). The methanolic extraction resulted in a more constant sensitivity (from 76 detected peaks to 67, in the same range). To estimate the sensitivity of each method, the average S/N was calculated for all common peaks (those detected by the 3 methods), resulting in a lower S/N ratio in all regions when the exosomal pellet was directly analyzed by ^1^H HR-MAS (Fig. 3).

In view of these data, its simplicity, and the lack of interference from solvents as chloroform, we chose the methanol method to investigate a role of the urinary exosomal metabolites in CVD after a proper reproducibility evaluation. One spot urine was divided into five aliquots and individually and equally processed to firstly isolate the exosomal fraction and then perform the metabolite extraction from each of the five exosomal pellets obtained, individually. Metabolite extracts from the five replicates were then analyzed by ^1^H NMR. The high reproducibility of the method can be seen in Additional file 3: Fig. S3, showing the five spectra obtained. The average number of peaks was 110 (93–132) (minimum-maximum) with a coefficient of variation of 13%. No significant difference was observed in peak intensity among the replicates (*p* value = 0.2451).

### Identification of a cardiometabolic signature in urinary exosomes: what exosomes add to urine analysis

**Fig. 4:**
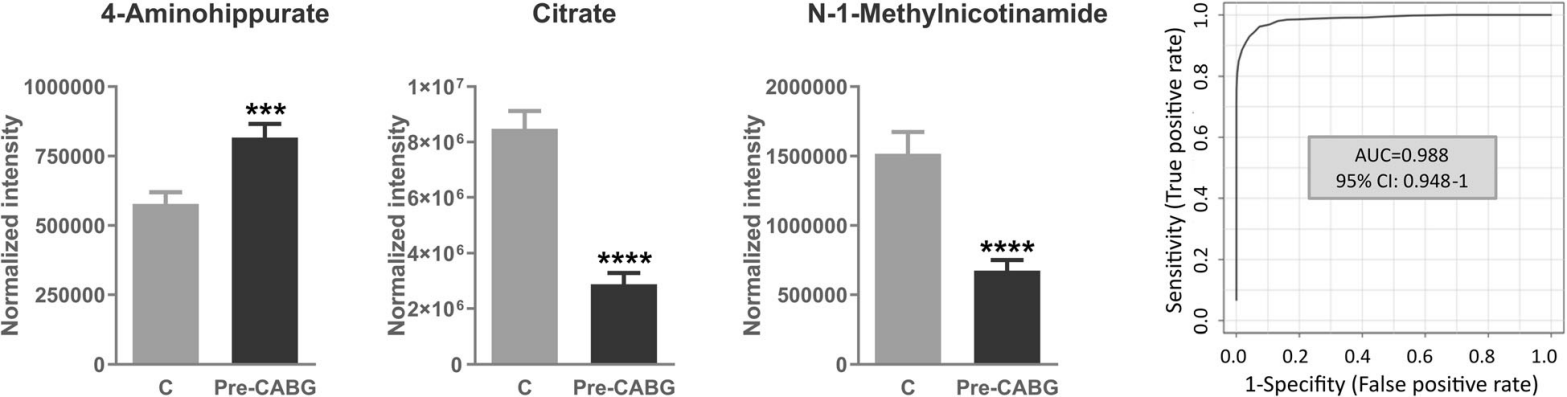
Target mass spectrometry analysis of exosomal metabolites showing variation between control subjects (*n* = 14) and CV risk patients (pre-CABG) (*n* = 18): 4-aminohippuric acid, citric acid, and N-1-methylnicotinamide. Receiver operating curve (ROC) resulting when the three metabolites are combined is shown. The Mann-Whitney test with 95% confidence level was applied. CABG coronary artery by-pass grafting. ***p value < 0.001, ****p value < 0.0001

With the aim of investigating a potential role for the metabolic content of urinary exosomal fraction in CVD, we recruited patients with programmed CABG as the CV risk group, and healthy donors without CV risk factors (hypertension, diabetes, or CVD previously documented, being that myocardial infarction, angina, or cardiac insufficiency). Clinical data are compiled in Table 1. Urine was collected, exosomes were isolated as described, and the metabolites were extracted following the methanol protocol. Metabolic exosomal extracts were analyzed by ^1^H NMR. Once isolated from urine, the exosomal pellets were weighted, resulting in no significant differences between the CV risk and control groups (Mann-Whitney non-parametric test, *p* value = 0.9840). Partial least square discriminant analysis (PLS-DA) of the exosomal metabolome shows good separation between the two study groups (Additional file 4: Fig. S4). Additional file 5: Table S1 shows those chemical shifts found with significant variation (*p* value < 0.05) and further analyzed by target MS (Additional file 6: Table S2). An exosomal metabolic fingerprint associated with CV risk was identified composed by 4-aminohippuric acid, N-1-methylnicotinamide, and citric acid. As can be seen in Fig. 4, 4-aminohippuric acid was found increased in the CV risk group, whereas citric acid and N-1-methylnicotinamide showed lower levels in that group. A permutation test confirmed the statistical significance for the three metabolites, resulting in *p* value < 0.0001 for N-1-methylnicotinamide and citric acid and *p* value = 0.0045 for 4-aminohippuric acid. To evaluate the clinical utility in terms of sensitivity and specificity, a combined ROC curve was calculated. Figure 4 shows the ROC resulting when the three metabolites (4-aminohippuric acid, N-1-methylnicotinamide, and citric acid) are considered.

**Table 1:** Clinical data of control subjects and patients undergoing coronary artery by-pass grafting (CABG) included in the study. *PVD* peripheral vascular disease

	Control subjects	CABG patients
Age	49 ± 6	68 ± 9
Sex (% male)	43	52
Diabetes (%)	0	26
Hypertension (%)	0	89
Previous coronary event (%)	0	56
Dyslipidemia	0	78
PVD	0	15

**Fig. 5:**
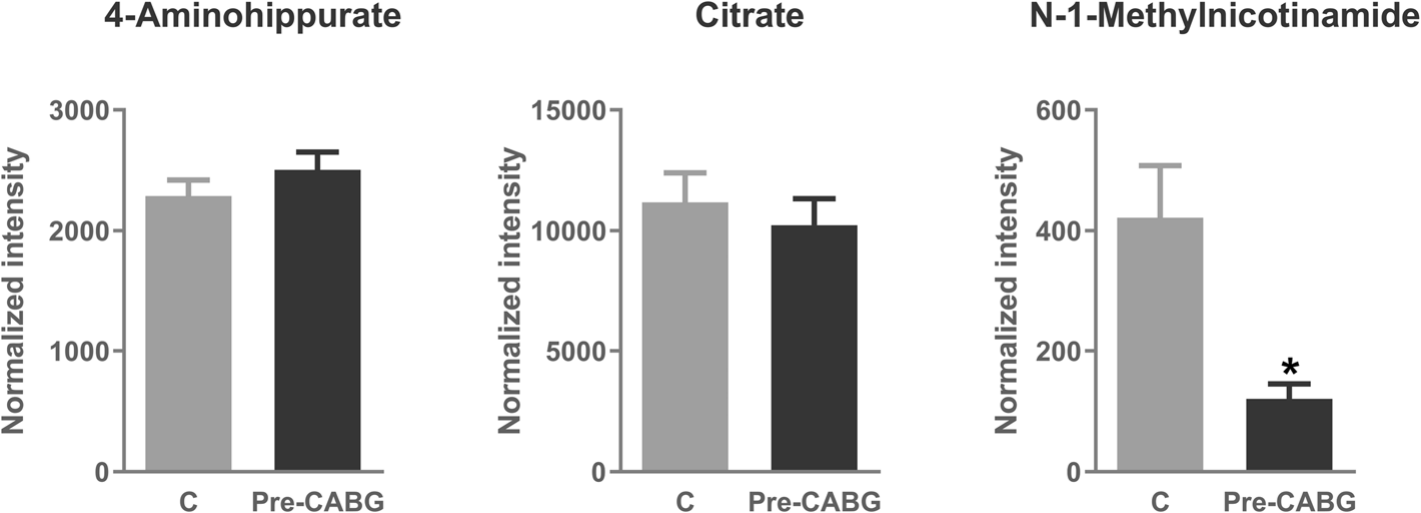
Target mass spectrometry analysis of urine metabolites in control (*n* = 24) and CV risk (*n* = 26) groups: 4-aminohippuric acid, citric acid, and N-1-methylnicotinamide. The Mann-Whitney test with 95% confidence level was applied. CABG coronary artery by-pass grafting. *p value < 0.05

To evaluate if the exosomes have added value to urine also at the metabolome level, we analyzed the identified metabolites directly in the urine of the same CABG patients (CV risk group) and control individuals by target MS. As can be seen in Fig. 5, 4-aminohippuric acid does not show variation between groups, contrary to what was observed when analyzed in the exosomal fraction. Citric acid tends to diminish but without significance, and the change in N-1-methylnicotinamide is less pronounced in urine than the one observed in the exosomes proving, thus, the added value of study urine exosomal metabolome.

## Discussion

CVD accounts for more than 30% of all global deaths. Despite enormous efforts on prevention, further research and novel indicators of CV risk are needed. Complementary to biological fluids, exosomes reflect molecular alterations that otherwise would be masked if measured directly in the biological fluids of origin. Their role as biomarker source or biological messengers in inter- and intracellular communication has been also evidenced [19]. Particularly, their study in CVD is gaining interest [20–23] but, to the best of our knowledge, there is no previous evidence on their metabolic content in this clinical context.

Up to date, the metabolome of the urinary exosomes is underexplored. There are only two studies on the metabolic analysis of urinary exosomes [24, 25], both by target LC-MS/MS and in prostate cancer. This study represents the first report of the analysis of the urinary exosomal metabolites by NMR and furthermore proposes a methodology compatible with both, NMR and LC-MS. Also, this is the first evidence of exosome metabolic alterations in CVD.

The comparison between extraction methods (methanol-based and chloroform-based) and non-extraction method (^1^H HR-MAS) shows higher S/N when the metabolites are extracted to liquid phase, which may be a consequence of the removal of interference compounds affecting ^1^H HR-MAS spectrum (e.g., lipids). Both extraction methods perform similarly in the high-field region (0–5 ppm), and the aromatic area is specially favored from the extraction procedure, particularly in the chloroform-based method. However, the chloroform itself constitutes an interference in that area, thus limiting the beneficial of chloroform-based extraction.

Three metabolites were identified in urinary exosomes from CV risk patients with an altered pattern compared to healthy individuals: 4-aminohippuric acid, N-1-methylnicotinamide, and citric acid. In atherosclerosis development, excess reactive oxygen species (ROS) causes arterial wall remodeling with smooth muscle cell proliferation and increased inflammation [26]. In our cohort of CV risk patients, increased production of ROS is expected. Higher levels of 4-aminohippuric acid found in these patients are in agreement with increased levels of this metabolite previously reported in conditions of higher ROS production and enhanced inflammatory response [27]. N-1-Methylnicotinamide has vasorelaxating properties and may exert antithrombotic and anti-inflammatory effects [28, 29]. Decreased levels found here in CV risk patients are in accordance with a potential protective role in the arterial wall, serving as a molecular indicator of atherosclerosis development. Our group previously reported tricarboxylic acid (TCA) cycle metabolic deregulation in hypertensive individuals of higher CV risk, e.g., albuminuria development or resistant hypertension [17]. Here, reduced levels of citric acid were found in CV risk patients, following the same trend previously observed in chronic kidney disease (CKD) versus healthy subjects with aggravated trend in those CKD patients with CVD [18].

One limitation of the study is the limited number of patients and control subjects included, which may compromise the conclusions due to unrelated sources of variation. Further studies should follow in wider cohorts for additional validation.

## Conclusions

A novel methodology to investigate metabolic alterations in urine at the exosomal level has been developed for NMR, compatible with LC-MS/MS analysis, and successfully applied here to clinical samples. This study constitutes the first evidence of a role for the exosomal metabolism in CVD and opens a new field of research in other pathologies. An exosomal metabolic signature linked to CVD was identified, confirming the existence of specific metabolite deregulation in urinary exosomes demonstrating the added value of extracellular vesicles in biomarker research.

## Methods

### Patient recruitment and urine collection

Human urine samples were collected in sterile containers and immediately transported to the laboratory. For exosome analysis, a minimum volume of 50 mL urine was collected and protease inhibitors cocktail was added (Sigma P8340). For urine metabolome analysis, 1 mL was centrifuged (16,200*g*, 10 min, 4 °C) and the supernatant was collected. Samples were stored at − 80 °C until analysis. A total of 27 patients undergoing coronary artery by-pass grafting (CABG) at Fundación Jiménez Díaz Hospital (Madrid, Spain) and 28 healthy subjects from the Donation Unit at the same hospital were recruited. The study was conducted according to the recommendations of the Declaration of Helsinki and was approved by the local ethics committee (PIC51-2013). In all cases, informed consent was requested indicating that their participation in the study was not prejudicial in any way to the treatment and possesses no risk.

### Urinary exosome isolation and characterization

Ultracentrifugation remains a mainstay for biomarker discovery in EVs [30, 31]. The exosome-like fraction (here referred as exosomes) was obtained from urine by modifying and adapting a previously published protocol [10, 11, 32]. Urine samples were defrosted and vigorously vortexed. Then, samples were centrifuged (17,000*g*, 10 min, 4 °C) to remove cell debris and the supernatant was collected. The pellet was treated with 200 mg/mL dithiothreitol (DTT) in PBS (150 mM NaCl, 12.5 mM Na_2_HPO_4_, 11.5 mM NaH_2_PO_4_, in ddH_2_O, pH 7.2), instead of isolation solution as previously published, and heated at 37 °C for 10 min in order to reduce Tamm-Horsfall protein (THP) networks and release trapped exosomes [33]. Then, 10 mL of PBS was added and a new low-speed centrifugation (17,000*g*, 10 min) was performed. Following centrifugation, the resulting supernatant was collected, mixed with the one obtained from the first centrifugation step, and finally ultracentrifuged (175,000*g*, 70 min, 4 °C) to pellet the exosomes. These modifications make the exosomes compatible with subsequent NMR and LC-MS analyses.

Isolated exosomes were characterized by electron microscopy by suspension in 4% paraformaldehyde in PBS and spotted on parafilm. A Formvar-carbon-coated grid (Ted Pella Inc. CA-USA) was floated on the sample droplet for 3 min and then on a drop of ddH_2_0 for 1 min. To obtain the contrast of the samples, a negative stain was carried out by floating the grid on a drop of 2% uranyl acetate in ddH_2_0 for 30 s. The excess fluid was removed, and the results were visualized using a JEM 1010 transmission microscope (JEOL Peabody, MA, USA). Additionally, exosomes were characterized by Western blot with primary antibodies Alix (1:500, Santa Cruz; (1A12) SC-53540 Lot: K1115) and TSG-101 (1:500, Abcam; [4A10] (ab83) Lot: GR3218062-2). Rabbit anti-mouse-HRP (1:2500, Santa Cruz) was used as a secondary antibody.

### Metabolite extraction from urinary exosomes

For metabolite extraction from urinary exosomes, two protocols were set up and compared. The first one was based on and improved from a protocol previously applied in the laboratory to aortic tissue [5]. The exosomal pellet was solubilized in 600 μL of cold methanol (MeOH):H_2_O (1:1), vigorously vortexed, and sonicated for 30 min at 4 °C. The samples were then centrifuged, and the supernatant was collected. The second protocol, described to extract metabolites from exosomes isolated from plasma and culture media [34], was here adapted and modified accordingly to be tested in urinary exosomes. For that, the exosome pellet was solubilized in 200 μL of ddH_2_O, left on ice for 30 s, heated at 37 °C for 90 s, and sonicated at 4 °C for 30 s. Then, 200 μL of cold MeOH was added followed by 200 μL of chloroform, and samples were centrifuged (16,200*g*, 10 min, 4 °C). The supernatant was transferred to a new tube, and 200 μL of acetonitrile (ACN) was added. Samples were incubated for half an hour at 4 °C and centrifuged, and the supernatant was stored at − 20 °C until further analysis.

### ^1^H HR-MAS analysis of metabolites from intact exosomal fraction: spectra acquisition

High-resolution magic angle spinning (^1^H HR-MAS) is a non-destructive technique which allows direct measure on tissue without extracting the metabolites. We tested it by direct analysis of the exosomal pellet without solubilization and operating at 4 °C to avoid sample degradation. ^1^H HR-MAS spectroscopy was performed at 500.13 MHz using a Bruker AVIII500 spectrometer 11.7 T. Samples were placed within a 50-μL zirconium oxide rotor with cylindrical insert and spun at 5000-Hz spinning rate, to remove the effects of spinning side bands from the spectra acquired. Standard solvent suppressed spectra were acquired into 32 k data points, averaged over 512 acquisitions, using a sequence based on the first increment of the NOESY pulse sequence. A spectral width of 6009 Hz was used.

A Carr-Purcell-Meibom-Gill (CPMG) pulse sequence was used as a T2 filter to suppress broad signals from macromolecules, so permitting the identification of small metabolites. Water suppression was achieved through irradiation of water signal during 2 s. The following parameters were used: 512 scans, a spectral width of 6009 Hz, and 32 k data points. Spectra were processed using TOPSPIN software, version 3.5 (Bruker Rheinstetten, Germany). Spectra were phased, baseline-corrected, and referenced to the TSP singlet at *δ* 0 ppm.

### ^1^H NMR analysis of extracted exosomal metabolites: spectrum acquisition

The supernatant containing the exosomal metabolites was speed-vacuum dried, and the resulting pellet was resuspended in 100 mM sodium phosphate buffer containing 0.01 mM sodium (3-trimethylsilyl)-2,2,3,3-tetradeuteriopropionate (TSP) for chemical shift referencing. The use of PBS in exosome isolation instead of other solvents or solutions is key for the compatibility with NMR approach. All NMR experiments were performed at 278 K on a Bruker 700-MHz AVANCE III instrument operating at 700.17 MHz equipped with a 5-mm triple resonance cryoprobe with a *z*-axis gradient. NMR spectra were acquired using a standard pulse sequence noesypr1d with water suppression during relaxation time 2 s, the acquisition time was 1.45 s, and the mixing time was 150 ms. The number of scans was 512 and a 90° pulse set to 14 μs, using 32 k data points with a spectral width of 16 ppm. Line broadening at 1 Hz was applied before Fourier transformation. All spectra were manually phased, baseline-corrected, and calibrated to TSP (*δ* 0.00 ppm) with TopSpin 3.2 (Bruker, Rheinstetten, Germany).

### Method performance comparison

Peak peaking and S/N calculation were performed by ACDLabs software, version 2019.1, and TopSpin software, version 3.5 (Bruker, Rheinstetten, Germany). Considering these data, statistical analysis using GraphPad Prism 6 software was performed to compare the three set-up protocols to study the exosome metabolome by NMR. To characterize the resultant spectra from the three methods, peak intensities recorded for each of them were classified in quartiles as follows: “high intensity” peaks, with intensity higher than the 3rd quartile cut-off value (≥ 75% of maximum intensity); “low intensity” peaks, with intensity lower than the 1st quartile cut-off value (≤ 25% of maximum intensity); and “medium intensity” peaks for those with intensity in between. Additionally, the average signal to noise ratio (S/N) was calculated, considering those peaks detected in the three methods with a S/*N* > 3. Finally, the robustness of the selected method (reproducibility) was further evaluated by analyzing five replicates (non-parametric Kruskal-Wallis test).

### Screening of exosomal metabolites showing alteration in CV risk patients by NMR

NMR spectra were analyzed using AMIX software (version 3.9.15, Bruker Rheinstetten, Germany). Each spectrum, from 0.5 to 10.00 ppm, was partitioned into equally sized spectral regions of 0.04 ppm (buckets) which were individually integrated by summing up the intensities of every experimental point inside each bucket. For normalization, individual bucket integrals so obtained were scaled by dividing their values by the total spectral integral, once excluding the water resonance region (4.75–5.25 ppm). As a first screening method, distribution of every bucket variable over the ensemble of spectra was evaluated by AMIX software. By Metaboanalyst 4.0 public web server [35], partial least square discriminant analysis (PLS-DA) was performed following normalization (90% confidence level, scaled to unit variance). Chemical shifts with fold change between clinical groups ≥ 3 and *p* value < 0.05 were selected. Metabolite annotations were carried out by using Chenomx NMR Suite 8.3 profiler (Chenomx NMR Suit 2017, Edmonton, Canada) and the HMBD database version 4.0 [36]. 2D NMR analysis was carried out to confirm the annotations by homonuclear correlation spectroscopy ^1^H–^1^H (COSY) and heteronuclear single-quantum correlation spectroscopy ^1^H–^13^C (HSQC).

### Exosomal metabolites target analysis by mass spectrometry

Exosomal metabolites with potential variation between CABG patients and healthy subjects were further analyzed by target mass spectrometry in SRM mode, coupled to liquid chromatography (SRM-LC-MS/MS) [5, 15]. In SRM, a specific precursor and its correspondent fragment ion (transition) were measured for every metabolite (Supplementary Table 2), previously determined by direct infusion of commercial standards. We used a 6460 Triple Quadrupole LC-MS/MS (1200 Series, Agilent Technologies) controlled by Mass Hunter Software (v4.0 Agilent Technologies). In a first quadrupole (Q1), the metabolite precursor is selected and, following fragmentation in Q2, the most intense fragment is selected in Q3. In this way, specific transitions (precursor --> fragment) were analyzed to quantify the metabolites of interest.

Metabolite extracts were speed-vacuum dried, and the metabolites were solved in 50% cold methanol, filtered (0.22 μm), and directly analyzed on an Atlantis T3 column (Waters) thermostatically controlled at 40 °C. A sample volume of 10 μL was injected and separation took place at 0.4 mL/min in an acetonitrile gradient: (1) at 0 min 0% B (0.1% formic acid in acetonitrile), (2) at 1 min 0% B, (3) at 2.5 min 95% B, (4) at 2.51 min 0% B, and (5) at 5 min 0% B. Dwell time was fixed to 50 ms and delta EMV to 400 V in positive mode and 600 V in negative one. Collision energy and fragmentor potential in the range 60–175 V were optimized for each metabolite by analyzing commercial metabolite standards in the previous set-up analysis. Additionally, to evaluate the complementary value of urinary exosome metabolome, urine analysis of those metabolites was performed following the same analytical conditions; urine proteins were removed by organic precipitation, and the supernatant was collected for analysis by SRM-LC-MS/MS.

To avoid potential bias in the exosomal recovery, the final pellets were weighted for every subject, and peak area values were normalized by the pellet weight. For urine analysis, metabolite signals were normalized by urinary creatinine (mg/dL). Groups were compared by non-parametric Mann-Whitney test with 95% confidence level (GraphPad Prism 6 software). The ROUT method was applied to detect outliers based on the false discovery rate, setting the *Q* value to 5%. Receiver operating characteristic (ROC) curves were calculated by means of Metaboanalyst web server (version 4.0) [35]. ROC curves were generated by Monte-Carlo cross-validation (MCCV) using balanced subsampling. In each MCCV, two thirds (2/3) of the samples are used to evaluate the feature importance. The top important features are then used to build classification models which are validated on 1/3 of the samples that were left out. This procedure was repeated multiple times to calculate the performance and confidence interval of each model. Random Forests was selected as the classification method and feature ranking built-in method.

To further confirm the statistical significance of the exosomal metabolites, a permutation test was performed. The null hypothesis that there is no difference between patients and controls  was simulated 10,000 times. For each of these simulated samples, the difference between the medians of patients and controls  was calculated. Finally, the proportion of these 10,000 differences that exceeds the difference of the original sample  was taken out, and it  was multiplied by two to make the test bilateral.

## Supplementary Information

**Additional file 1: Figure S1.** Characterization of the three tested exosomal NMR workflows in terms of detected signals per spectral region.

**Additional file 2: Figure S2.** Evaluation of the sensitivity (S/N ratio) of the three tested exosomal NMR protocols.

**Additional file 3: Figure S3.** Reproducibility analysis of the selected NMR method based on MeOH extraction.

**Additional file 4:** **Figure S4.** Partial least square discriminant analysis (PLS-DA) of the exosomal metabolome between control and CV risk patients (Pre-CABG).

**Additional file 5: Table S1.** Statistically significant chemical shifts from exosomal NMR comparative analysis.

**Additional file 6: Table S2.** Technical conditions for SRM-LC-MS/MS analysis of exosomal and urine metabolome.

**Additional file 7: Table S3.** Raw data for NMR and LC-MS/MS analyses.

## Data Availability

The datasets used and/or analyzed during the current study are included in this published article and its supplementary information files.

## Abbreviations

ACN: Acetonitrile
AUC: Area under the roc curve
CABG: Coronary artery by-pass grafting
CKD: Chronic kidney disease
COSY: Homonuclear correlation spectroscopy ^1^H–^1^H
CV: Cardiovascular
CVD: Cardiovascular disease
DTT: Dithiothreitol
^1^H HR-MAS: High-resolution magic angle spinning
HSQC: Heteronuclear single-quantum correlation spectroscopy ^1^H–^13^C
LC-MS/MS: Mass spectrometry coupled to liquid chromatography
MeOH: Methanol
NMR: Nuclear magnetic resonance
PLS-DA: Partial least square discriminant analysis
ROC: Receiver operating characteristic curve
ROS: Reactive oxygen species
S/N: Signal to noise ratio
SRM: Selected reaction monitoring
TCA: Tricarboxylic acid
THP: Tamm-Horsfall protein
TSP: Sodium trimethylsilyl propionate
