# Supplementary material for: Analysis of urinary exosomal metabolites identifies cardiovascular risk signatures with added value to urine analysis
Source: BMC Biol. 2020 Dec 14;18:192. doi: 10.1186/s12915-020-00924-y (PMC7737341; doi:10.1186/s12915-020-00924-y)
Supplement: Supplementary file 2 — Additional file 2: Figure S2. Evaluation of the sensitivity (S/N ratio) of the three tested exosomal NMR protocols. [file 12915_2020_924_MOESM2_ESM.pptx]

## Slide 1
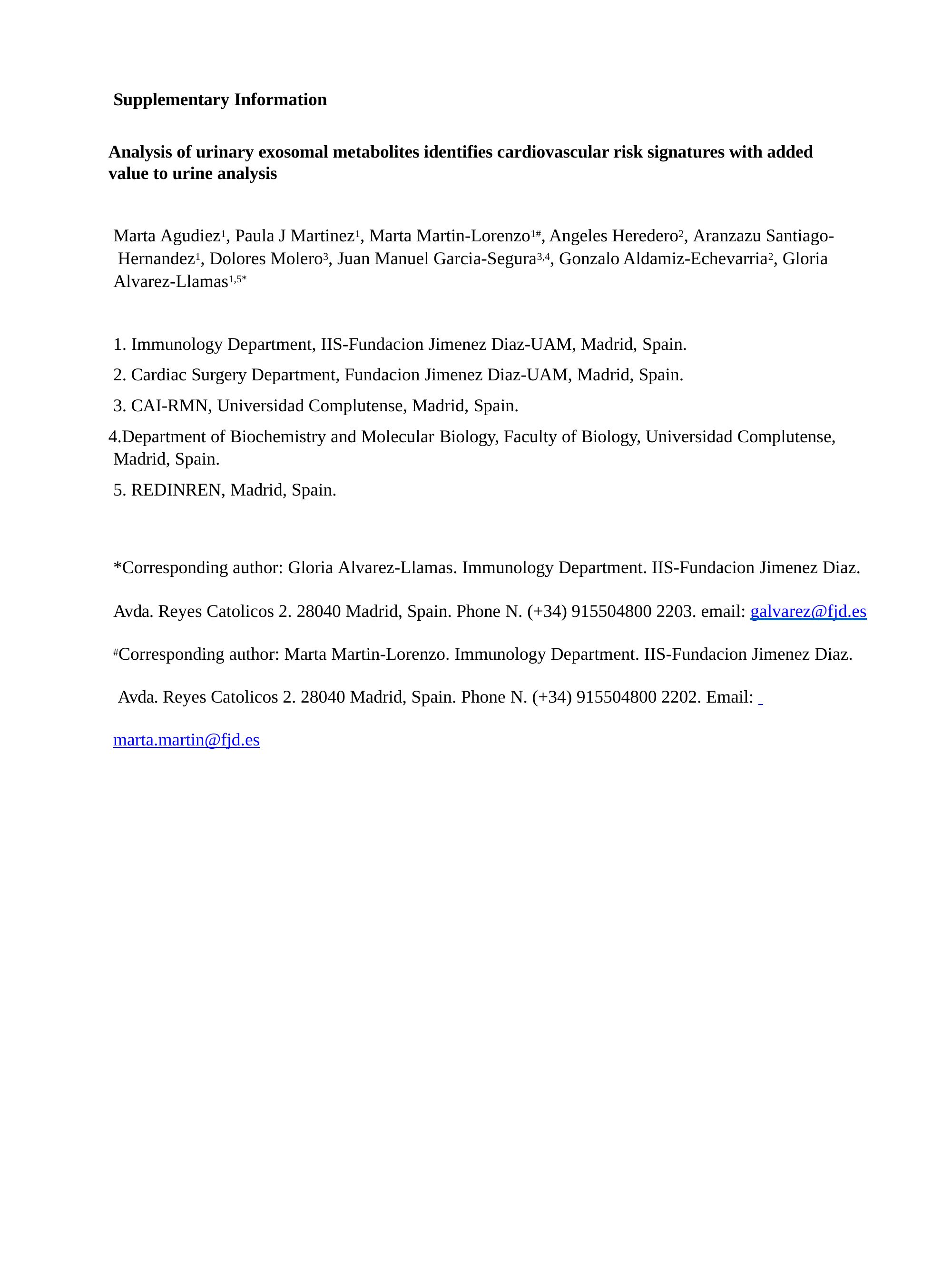

Supplementary Information
Analysis of urinary exosomal metabolites identifies cardiovascular risk signatures with added value to urine analysis
Marta Agudiez1, Paula J Martinez1, Marta Martin-Lorenzo1#, Angeles Heredero2, Aranzazu Santiago- Hernandez1, Dolores Molero3, Juan Manuel Garcia-Segura3,4, Gonzalo Aldamiz-Echevarria2, Gloria Alvarez-Llamas1,5*
Immunology Department, IIS-Fundacion Jimenez Diaz-UAM, Madrid, Spain.
Cardiac Surgery Department, Fundacion Jimenez Diaz-UAM, Madrid, Spain.
CAI-RMN, Universidad Complutense, Madrid, Spain.
Department of Biochemistry and Molecular Biology, Faculty of Biology, Universidad Complutense, Madrid, Spain.
REDINREN, Madrid, Spain.
*Corresponding author: Gloria Alvarez-Llamas. Immunology Department. IIS-Fundacion Jimenez Diaz.
Avda. Reyes Catolicos 2. 28040 Madrid, Spain. Phone N. (+34) 915504800 2203. email: galvarez@fjd.es
#Corresponding author: Marta Martin-Lorenzo. Immunology Department. IIS-Fundacion Jimenez Diaz. Avda. Reyes Catolicos 2. 28040 Madrid, Spain. Phone N. (+34) 915504800 2202. Email: marta.martin@fjd.es

## Slide 2
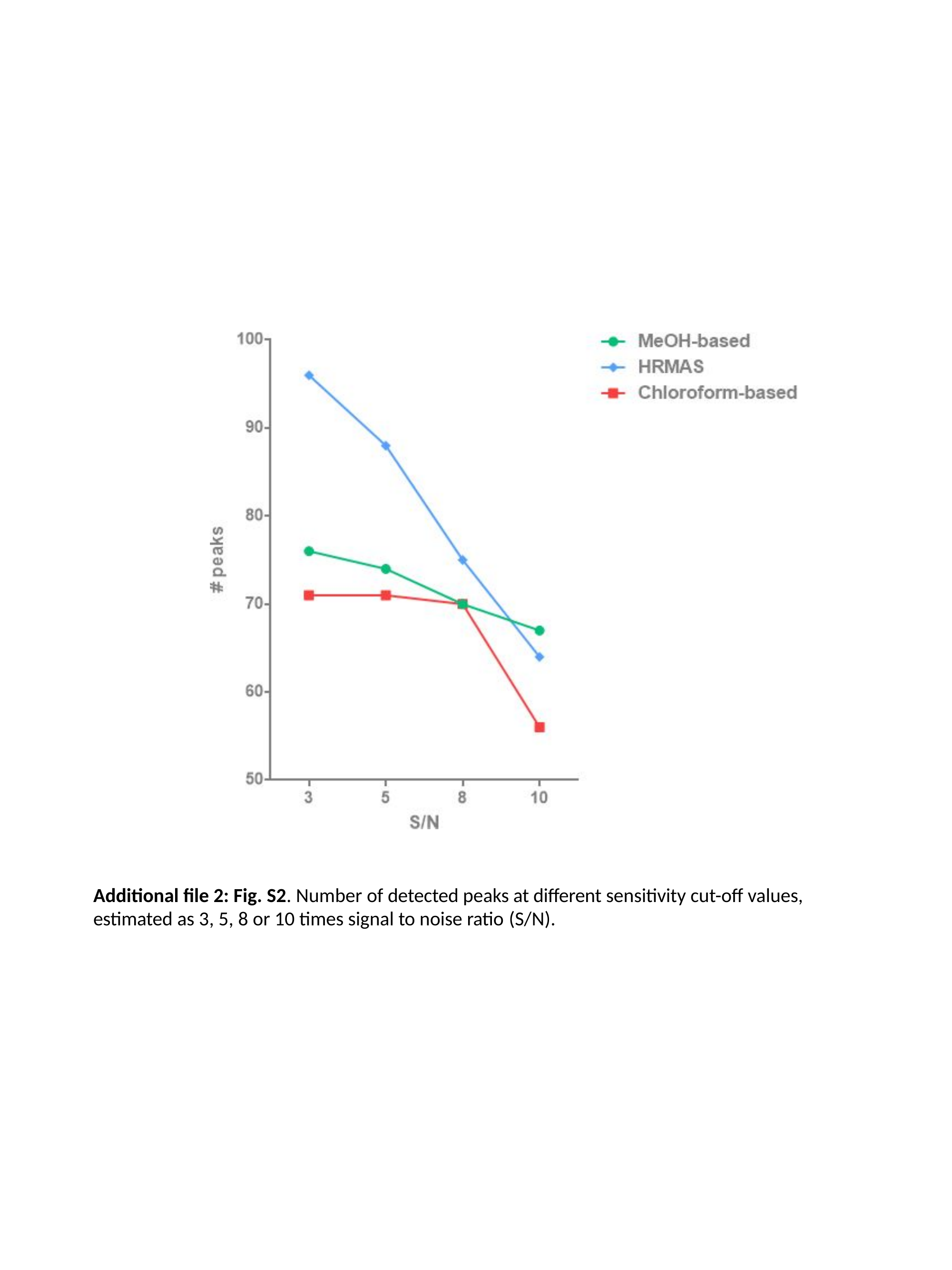

Additional file 2: Fig. S2. Number of detected peaks at different sensitivity cut-off values, estimated as 3, 5, 8 or 10 times signal to noise ratio (S/N).
