# Supplementary material for: Analysis of urinary exosomal metabolites identifies cardiovascular risk signatures with added value to urine analysis
Source: BMC Biol. 2020 Dec 14;18:192. doi: 10.1186/s12915-020-00924-y (PMC7737341; doi:10.1186/s12915-020-00924-y)
Supplement: Supplementary file 5 — Additional file 5: Table S1. Statistically significant chemical shifts from exosomal NMR comparative analysis. [file 12915_2020_924_MOESM5_ESM.docx]

**Supplementary Information**

**Analysis of urinary exosomal metabolites identifies cardiovascular risk signatures with added value to urine analysis**

Marta Agudiez^1^, Paula J Martinez^1^, Marta Martin-Lorenzo^1#^, Angeles Heredero^2^, Aranzazu Santiago-Hernandez^1^, Dolores Molero^3^, Juan Manuel Garcia-Segura^3,4^, Gonzalo Aldamiz-Echevarria^2^, Gloria Alvarez-Llamas^1,5*^

1. Immunology Department, IIS-Fundacion Jimenez Diaz-UAM, Madrid, Spain.

2. Cardiac Surgery Department, Fundacion Jimenez Diaz-UAM, Madrid, Spain.

3. CAI-RMN, Universidad Complutense, Madrid, Spain.

4. Department of Biochemistry and Molecular Biology, Faculty of Biology, Universidad Complutense, Madrid, Spain.

5. REDINREN, Madrid, Spain.

*Corresponding author: Gloria Alvarez-Llamas. Immunology Department. IIS-Fundacion Jimenez Diaz. Avda. Reyes Catolicos 2. 28040 Madrid, Spain. Phone N. (+34) 915504800 2203. email: [galvarez@fjd.es](mailto:galvarez@fjd.es)

^#^Corresponding author: Marta Martin-Lorenzo. Immunology Department. IIS-Fundacion Jimenez Diaz. Avda. Reyes Catolicos 2. 28040 Madrid, Spain. Phone N. (+34) 915504800 2202. Email: marta.martin@fjd.es

**Additional file 5: Table S1.** Exosomal metabolites showing differential abundance in urine from coronary artery by-pass surgery (CABG) patients (CVrisk group) versus healthy subjects (control group) by nuclear magnetic resonance (NMR). Experimental and matched theoretical chemical shifts are shown (HMDB).

| **Metabolite** | **Experimental chemical shift**  **^1^H NMR (ppm)** | **Theoretical chemical shift**  **^1^H NMR (ppm)** | **p-value** |
| --- | --- | --- | --- |
| 4-Aminohippuric acid | 6.841 | 6.852 | 0.0099 |
| N-1-Methylnicotinamide | 4.448  8.154  8.902 | 4.475  8.179  8.892 | 0.0109  0.0082  0.0470 |
| Citric acid | 2.540 | 2.530 | 0.0254 |
