# Supplementary material for: Analysis of urinary exosomal metabolites identifies cardiovascular risk signatures with added value to urine analysis
Source: BMC Biol. 2020 Dec 14;18:192. doi: 10.1186/s12915-020-00924-y (PMC7737341; doi:10.1186/s12915-020-00924-y)
Supplement: Supplementary file 6 — Additional file 6: Table S2. Technical conditions for SRM-LC-MS/MS analysis of exosomal and urine metabolome. [file 12915_2020_924_MOESM6_ESM.docx]

**Supplementary Information**

**Analysis of urinary exosomal metabolites identifies cardiovascular risk signatures with added value to urine analysis**

Marta Agudiez^1^, Paula J Martinez^1^, Marta Martin-Lorenzo^1#^, Angeles Heredero^2^, Aranzazu Santiago-Hernandez^1^, Dolores Molero^3^, Juan Manuel Garcia-Segura^3,4^, Gonzalo Aldamiz-Echevarria^2^, Gloria Alvarez-Llamas^1,5*^

1. Immunology Department, IIS-Fundacion Jimenez Diaz-UAM, Madrid, Spain.

2. Cardiac Surgery Department, Fundacion Jimenez Diaz-UAM, Madrid, Spain.

3. CAI-RMN, Universidad Complutense, Madrid, Spain.

4. Department of Biochemistry and Molecular Biology, Faculty of Biology, Universidad Complutense, Madrid, Spain.

5. REDINREN, Madrid, Spain.

*Corresponding author: Gloria Alvarez-Llamas. Immunology Department. IIS-Fundacion Jimenez Diaz. Avda. Reyes Catolicos 2. 28040 Madrid, Spain. Phone N. (+34) 915504800 2203. email: [galvarez@fjd.es](mailto:galvarez@fjd.es)

^#^Corresponding author: Marta Martin-Lorenzo. Immunology Department. IIS-Fundacion Jimenez Diaz. Avda. Reyes Catolicos 2. 28040 Madrid, Spain. Phone N. (+34) 915504800 2202. Email: marta.martin@fjd.es

**Additional file 6: Table S2.** Instrumental conditions for metabolites target analysis by SRM-LC-MS/MS.

| **Metabolite** | **Precursor mass (m/z)** | **Fragment mass (m/z)** | **Dwell time (s)** | **Fragmentor voltage (V)** | **Collision energy** | **Mode** |
| --- | --- | --- | --- | --- | --- | --- |
| 4-Aminohippuric acid | 265.91 | 45.9 | 50 | 130 | 30 | + |
| 1-Methylnicotinamide | 138.2 | 93.1 | 50 | 100 | 26 | + |
| Citric acid | 191.1 | 86.9 | 50 | 120 | 14 | - |
